# Common Physical Performance Tests for Evaluating Health in Older Adults: Cross-Sectional Study

**DOI:** 10.2196/53304

**Published:** 2024-11-29

**Authors:** Chitra Banarjee, Renoa Choudhury, Joon-Hyuk Park, Rui Xie, David Fukuda, Jeffrey Stout, Ladda Thiamwong

**Affiliations:** 1 College of Medicine University of Central Florida Orlando, FL United States; 2 Department of Biomedical Engineering and Mechanics Virginia Tech Blacksburg, VA United States; 3 Department of Mechanical Engineering University of Central Flordia Orlando, FL United States; 4 Disability, Aging and Technology Cluster University of Central Florida Orlando, FL United States; 5 Department of Statistics and Data Science University of Central Florida Orlando, FL United States; 6 College of Nursing University of Central Florida Orlando, FL United States; 7 School of Kinesiology and Rehabilitation Sciences College of Health Professions and Sciences University of Central Florida Orlando, FL United States

**Keywords:** functional capacity, physical activity, fear of falling, physical performance tests, Short Physical Performance Battery, 6-minute walk test, Incremental Shuttle Walk Test, geriatrics, aging

## Abstract

**Background:**

Interdisciplinary evaluation of older adults’ health care is a priority in the prevention of chronic health conditions and maintenance of daily functioning. While many studies evaluate different physical performance tests (PPTs) from a retrospective view in predicting mortality or cardiopulmonary health, it remains unclear which of the commonly used PPTs is the most effective at evaluating the current health of older adults. Additionally, the time and participant burden for each PPT must be considered when planning and implementing them for clinical or research purposes.

**Objective:**

This cross-sectional study aimed to determine how elements of overall physical capacity, performance, and other nongait factors in older adults affect the results of 3 commonly used tests: the Short Physical Performance Battery (SPPB), 6-minute walk test (6MWT), and Incremental Shuttle Walk Test (ISWT).

**Methods:**

A total of 53 community-dwelling older adults met the inclusion and exclusion criteria (mean age 77.47, SD 7.25 years; n=41, 77% female; and n=21, 40% Hispanic). This study evaluated older adults using 3 different PPTs including the SPPB, 6MWT, and ISWT, as well as constructed multiple linear regression models with measures of physical activity, static balance, and fear of falling (FoF). The nongait measures included 7 days of physical activity monitoring using the ActiGraph GT9X Link instrument, objective measurement of static balance using the BTrackS Balance System, and FoF using the short Fall Efficacy Scale-International.

**Results:**

The models revealed that the complete SPPB provided the most comprehensive value, as indicated by a greater *R*^2^ value (0.523), and that performance on the SPPB was predicted by both moderate to vigorous physical activity (*P=*.01) and FoF (*P*<.001). The ISWT was predicted by moderate to vigorous physical activity (*P*=.02), BMI (*P*=.02), and FoF (*P*=.006) and had a similar *R*^2^ value (0.517), whereas the gait component of the SPPB (*P=*.001) and 6MWT (*P<*.001) was predicted by only FoF and had lower *R*^2^ values (0.375 and 0.228, respectively).

**Conclusions:**

The results indicated the value of a multicomponent, comprehensive test, such as the SPPB, in evaluating the health of older adults. Additionally, a comparison of the 2 field walking tests (ISWT and 6MWT) further distinguished the ISWT as more responsive to overall health in older adults. In comparing these commonly used PPTs, clinicians and researchers in the field can determine and select the most optimal test to evaluate older adults in communities and research settings.

## Introduction

Older adults are a rapidly increasing population that experiences increased chronic health conditions [1] and decreased daily functioning [2] compared to the general population. Physical activity is a key intervention recommended by health care providers to decrease the incidence of chronic health conditions, increase the ability to perform activities of daily living, and improve overall quality of life [3-5]. As such, evaluating physical capacity and health is of critical importance in older adults. Various physical performance tests (PPTs) have been shown to be important predictors of mortality [6]; however, few studies have compared the relevance of different PPTs to older adults’ physical activity, static balance, BMI, and fear of falling (FoF) within the same population of older adults. Here, this study aims to determine the PPT that is the most affected by overall physical capacity, performance, and other factors in older adults by comparing 3 commonly used tests (the Short Physical Performance Battery [SPPB], 6-minute walk test [6MWT], and Incremental Shuttle Walk Test [ISWT]).

Decreased physical performance in older adults has been associated with an increased risk of cardiovascular disease, physiological fall risk, and earlier onset of mental health conditions [7-9]. Cardiovascular disease is the leading cause of death in the United States [10]. For older adults specifically, declines in cardiovascular health have been shown to lead to further disability [7], highlighting the need for early detection using tests that are easy to perform. Time spent in moderate to vigorous physical activity (MVPA) is a cardioprotective measure associated with a lower incidence of cardiovascular disease and coronary heart disease in older adults [11,12]. However, measuring physical activity objectively requires equipment such as accelerometers or movement trackers. Similarly, 30% to 40% of older adults experience a fall yearly [13]. Identifying people at higher risk of falling has both social and financial incentives, as falls exacerbate existing health conditions, further decrease the quality of life for older adults, and cause injuries that pose a significant financial burden for the health care system [14]. Fall risk is assessed objectively by devices that test static balance and subjectively through questionnaires such as the short Fall Efficacy Scale-International (FES-I). Measuring both the physiological and perceived fall risk can help us understand the incongruence between balance performance and FoF in older adults to inform targeted fall prevention strategies [15]. Finally, physical performance has also been associated with mental health including earlier onset of depression in older adults with poor performance. However, most of these studies that outline the implications of changes in physical performance were performed using different tests in different populations, often relying on longitudinal or retrospective data. Given these implications, the tools used for the assessment of physical activity and fall risk are of paramount importance [16,17].

Three PPTs—the SPPB, 6MWT, and ISWT—are compared in their response to physical activity levels, static balance, and FoF in older adults. Each of these PPTs is commonly used by physicians and health care personnel in evaluating the physical fitness of older adults and recovering patients [18-20]. The SPPB is a PPT of lower mobility that has been shown to be associated with physical activity, static balance, and FoF [21-23]. The SPPB is composed of 3 different parts, including a gait speed component. The 6MWT is a gait measure associated with static balance and FoF [24,25]. Similarly, the ISWT is a more recent gait-related measure conducted incrementally and associated with physical activity and dynamic balance [26,27]. While the SPPB has been widely used as a measure of overall physical function and frailty, the use of 6MWT and ISWT has been predominantly limited to characterizing cardiopulmonary functional capacity and shown to be predictive of outcomes for patients with heart failure [28-30]. Some work has investigated the differences between the specified PPTs; however, this mainly consists of meta-analyses comparing multiple studies or studies that focus on the predictive value of the measure in health outcomes [6,31,32]. These PPTs were selected for their widespread use and similar purposes; by comparing their effectiveness when modeled by nongait components of physical health, we aim to determine which provides the most comprehensive value for the time and methods involved in each. As more studies are conducted on the aging population, researchers are likely to use one of the abovementioned PPTs to evaluate physical capacity and well-being in clinical and nonclinical older adults. By evaluating the more holistic SPPB alongside the gait component of SPPB, the 6MWT, and the ISWT, this study aimed to determine how the 3 PPTs are affected by overall physical capacity, performance, and other nongait factors in older adults.

## Methods

### Overview

In this cross-sectional study, 65 participants were recruited from Central Florida using word of mouth, announcements, and flyers. Participants were selected using purposive sampling, based on the following inclusion criteria: (1) aged 60 years or older, (2) being able to walk with or without an assistive device (but without the assistance of another person), (3) fluency in English or Spanish, and (4) living in their own homes or apartments. The exclusion criteria were (1) having a medical condition that may preclude participation in balance tests (eg, inability to stand on the balance plate) and physical activity (eg, shortness of breath, dizziness, and tightness in the chest or unusual fatigue at light exertion), (2) currently receiving treatment from a rehabilitation facility, and (3) having medical implants (eg, pacemakers).

### Data Collection and Procedure

This study required 2 visits to the study site, and the data were collected from September 2022 to March 2023. In the first visit, participants completed a demographic survey and anthropometric measurements, followed by assessments of FoF and static balance. Then, participants completed the PPTs, including the SPPB, 6MWT, and ISWT.

At the end of the first visit, each participant was provided a wrist-worn accelerometer and wore it for 7-day physical activity monitoring in free-living conditions. On the second visit, participants returned the accelerometer and were compensated with a US $30 store gift card for their participation in the study.

### Ethical Considerations

The study was approved by the Institutional Review Board at the University of Central Florida (protocol STUDY00004365). All participants provided written informed consent to participate. The data have been anonymized and deidentified. Participants were compensated with a US $30 gift card for their participation in the study.

### Assessments

#### PPTs

For each of the following PPTs, trained research assistants demonstrated the procedure to the participant prior to the participant performing the test.

#### SPPB

The SPPB comprised 3 tests, which were performed in the following order: the standing balance test, the gait speed test, and the chair stand test [33].

The standing balance test took place in a quiet area with no visual distractions. Participants were instructed to wear comfortable footwear with a heel less than 3 cm (~1 inch) while performing three balance tests: (1) side-by-side stand: the participant attempted to stand with feet together, side-by-side, for 10 seconds; (2) semitandem stand: the participant attempted to stand with the side of the heel of one foot touching the big toe of the other foot for 10 seconds (participants were informed to start with the foot of their preference as per protocol [28]); and (3) tandem stand: the participant attempted to stand with the heel of one foot in front of and touch the toes of the other foot for about 10 seconds.

In the gait speed test, participants walked along a 4-meter course at their usual walk speed (ie, the pace walked from one place to another without urgency such as down a street or hallway). Participants were permitted to use assistive devices if required and completed the walk 2 times, for which the fastest attempt was recorded for scoring.

In the chair stand test, the participant was instructed to rise from a chair and sit down 5 times, as quickly as possible, without using their arms, and recorded the time required to complete the 5 chair stands. For each test, the experimenters demonstrated the task and guided the participant to the appropriate position, after which they started the test. Additionally, if the participant could not continue with the task for any reason, the test was terminated.

#### 6MWT

Participants walked along a flat, straight corridor of 12 meters in length, back and forth, as many times as they could for 6 minutes at their own pace. Participants were encouraged every 60 seconds using standardized phrasing. After 6 minutes, the total distance walked was recorded [34].

#### ISWT

A 10-meter course was marked with 2 cones. Upon reaching the second cone, the participant turned around, as modeled by the experimenter. Audio recordings of standardized instructions were played for participants to indicate walking speed. Each time the participant reached a cone was considered as 1 shuttle (ie, representing a 10-meter distance) and every single bleep from the audio recording signaled the end of a shuttle. The walking speed was progressively increased every minute, indicated by a triple bleep, and the test ended when the participant was unable to reach the turnaround points within the required time [35].

#### Physical Activity

The participants were instructed to wear the ActiGraph GT9X Link instrument (ActiGraph LLC) on their nondominant wrists for 7 consecutive days in a free-living environment. The ActiGraph accelerometer has been validated for assessing free-living physical activity [36]. After 7 days, the accelerometers were removed, and the raw data were downloaded and processed in R statistical software (R Core Team) using the *GGIR* package [37].

The data processing steps included (1) the autocalibration of acceleration signals according to local gravity [38]; (2) nonwear time detection; and (3) the calculation of the Euclidean norm (ie, vector magnitude) of acceleration minus 1 g, expressed in milli-gravitational units or mg (described as the sum of the squared acceleration components [Euclidean norm] minus 1 g). The time periods spent in sedentary behavior (SB), light-intensity physical activity (LPA), and MVPA were estimated using the following nondominant wrist–specific Euclidean norm minus 1 g cutoff points for older adults, adopted from the literature: (1) SB<30 mg, (2) 30 mg≤LPA<100 mg, and (3) MVPA≥100 mg [38-41]. Participants with at least 4 valid days were included in the analysis, and only days during which the accelerometer was worn for at least 14 hours were counted as valid days [38].

#### Static Balance

Static balance was assessed using the BTrackS Balance System (BBS). The BBS includes a portable BTrackS Balance Plate (BBP) and BTrackS Assess Balance software running on a computer device. The BBP’s dimensions were 15.5 × 23.5 × 2.5 in, weighing 6.58 kg (approved by the Food and Drug Administration). The participant stood as still as possible on the BBP for four 20-second trials. The software produced a normalized score using the BBS database to evaluate the performance of others of the same age and sex [42].

#### FoF

FoF was assessed using the short FES-I, which consists of a total of 7 items (eg, going in or out of a chair) on a 4-point scale, measuring concerns about the possibility of falling when performing activities (eg, getting dressed), which have been validated for community-dwelling older adults [43,44]. Total scores range from 7 to 28, where higher total scores indicate higher FoF [45]. Participants completed the short FES-I in a quiet and private room prior to PPT testing.

#### BMI

Height and weight were measured by experimenters using a stadiometer, and BMI was calculated by dividing the weight (kg) by the square of the height (m^2^) [46].

### Data Analysis

All statistical analyses were performed in R statistical software (version 4.3.0). Pearson correlation coefficient is appropriate for linear relationships, assumes normality, and achieves efficiency when linearity and normality assumptions are met, whereas Spearman rank correlation coefficient is suitable for nonlinear relationships, ordinal or nonnormally distributed data, and is robust to outliers [47]. As such, the correlation analysis was performed using the Spearman correlation coefficient to maintain validity for both normally distributed and nonnormally distributed variables. The correlation matrix was produced using the R package *corrplot*. The normality of variables was tested using the Shapiro-Wilks test. The effects of race and sex on PPT results were tested separately using ANOVA and Kruskal-Wallis tests, for normal and nonnormally distributed variables, respectively.

Multiple linear regression analysis was conducted to investigate the relationship between each PPT (SPPB, ISWT, and 6MWT) as outcome variables and the following predictors: physical activity (MVPA), static balance, FoF, BMI, and age. A priori sample size calculation for multiple linear regression revealed that the minimum number of samples for 5 explanatory variables at a statistical power level of 0.8, α=.05, and large effect size (Cohen *f*^2^=0.35) would be 43; therefore, our sample size (ie, N=53) had sufficient statistical power for multiple regression.

## Results

### Overview

This study included 53 older adults (mean age 77.47, SD 7.25 years) who were 77% (n=41) female. The sample included 5 (9%) African American, 21 (40%) Hispanic, 26 (49%) White, and 1 (2%) other race older adults.

### Correlation Analysis

**Figure 1:**
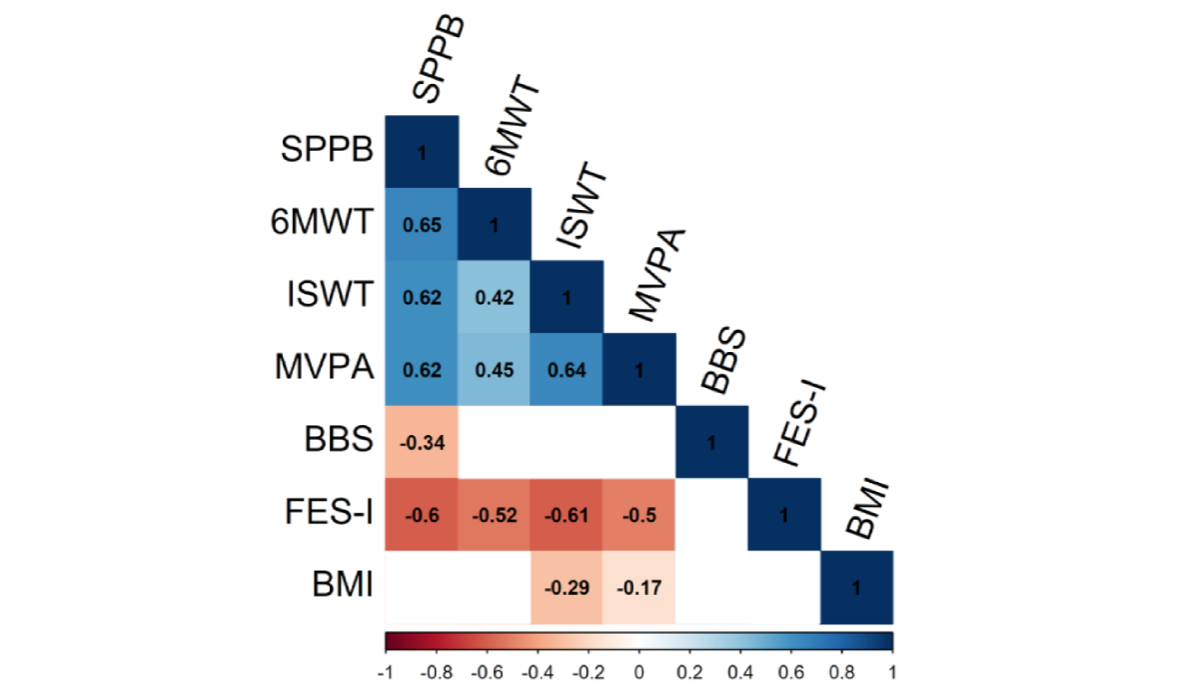
Correlation matrix between all collected and analyzed measures of physical performance tests. All values are significant (*P*<.05) Spearman correlation coefficients. Blank cells indicate insignificant relationships (*P*>.05). 6MWT: 6-minute walk test; BBS: BTrackS Balance System; FES-I: Fall Efficacy Scale-International; ISWT: Incremental Shuttle Walk Test; MVPA: moderate to vigorous physical activity; SPPB: Short Physical Performance Battery.

Spearman correlation coefficients for measures of physical activity (SB, LPA, and MVPA) yielded significant correlations with all measures (*P*<.05), except static balance (BBS) and BMI. Among the PPTs, the SPPB showed significant correlations with all measures, except BMI; the ISWT was correlated with all measures except static balance; and the 6MWT was correlated with all except LPA, static balance, BMI, and age. FoF (FES-I) was correlated with all measures except static balance and BMI. Static balance and BMI were both only correlated with a few measures (MVPA, SPPB, and ISWT), and age was correlated with all except 6MWT, static balance, and BMI (Figure 1).

### Effects of Sex and Race

The effects of sex and race were tested separately with ANOVA test and Kruskal-Wallis test for nonparametric variables, to evaluate differences in performance on the 3 PPTs, as shown in Table 1. For sex, the female group (n=41) was compared to the male group (n=12), finding no differences in the SPPB, 6MWT, or ISWT (*P*>.05; Table 1). Similarly, for race, the African American (n=5), Hispanic (n=21), and White (n=26) groups were compared, finding no differences in the SPPB, 6MWT, or ISWT (*P*>.05; Table 1). “Other” race was excluded in the tests as the sample size was 1. Based on these nonsignificant differences, sex and race were not included as covariates in the multivariable regression models below.

**Table 1:** ANOVA and Kruskal-Wallis tests comparing performance on PPT^a^ by sex and by race.

Demographics effect and PPT		Test	*P* value
**Sex^b^**			
	SPPB^c^	Kruskal-Wallis	.09
	6MWT^d^	ANOVA	.34
	ISWT^e^	ANOVA	.05
**Race^f^**			
	SPPB	Kruskal-Wallis	.47
	6MWT	ANOVA	.48
	ISWT	ANOVA	.42

^a^PPT: physical performance test.

^b^Female (n=41) and male (n=12).

^c^SPPB: Short Physical Performance Battery.

^d^6MWT: 6-minute walk test.

^e^ISWT: Incremental Shuttle Walk Test.

^f^African American (n=5), Hispanic (n=21), and White (n=26).

### Normality

The Shapiro-Wilks tests revealed that 4 of the 9 variables (SPPB, MVPA, static balance, and FoF) were not normally distributed.

### Multivariable Linear Regression Models

The multivariable regression models were used on each PPT (SPPB, ISWT, and 6MWT), as well as the gait component of SPPB (SPPB-G; 4-meter gait speed test) to relate them to the following predictors: physical activity (MVPA), static balance, FoF, BMI, and age (years).

Performance on the SPPB was significantly predicted by MVPA and the FES-I (Table 2). Performance on the 6MWT was significantly predicted solely by the FES-I (Table 3). Performance on the ISWT was significantly predicted by MVPA, BMI, and the FES-I (Table 4). Performance on the SPPB-G was similar to the 6MWT in that it was only predicted by the FES-I (Table 5). Supplemental analyses removing the FES-I as a covariate in the multivariable models showed no significant predictors for the 6MWT; however, the SPPB was then predicted by both MVPA and static balance, and the ISWT, as well as the SPPB-G, was predicted by MVPA, BMI, and age (Multimedia Appendix 1). Among the 3 models, the adjusted *R*^2^ value for the SPPB (0.523; Table 2) was greater than those for the 6MWT (0.228), ISWT (0.517), and SPPB-G (0.375). The models without the FES-I as a predictor showed decreased adjusted *R*^2^ values (all <0.500).

**Table 2:** Short Physical Performance Battery multivariable regression model.

Predictors^a^	β	95% CI	η^2^	*P* value
(Intercept)	12.94	5.86 to 20.02	N/A^b^	.001
MVPA^c^	.02	0.00 to 0.03	0.40	.01
BBS^d^	–.02	–0.04 to 0.01	0.14	.15
Short FES-I^e^	–.36	–0.53 to –0.20	0.33	<.001
BMI	.02	–0.09 to 0.12	0.417	.75
Age	–.01	–0.08 to 0.06	0.201	.76

^a^*R*^2^/*R*^2^ adjusted: 0.569/0.523.

^b^Not applicable.

^c^MVPA: moderate to vigorous physical activity.

^d^BBS: BTrackS Balance System.

^e^FES-I: Fall Efficacy Scale-International.

**Table 3:** 6-minute walk test multivariable regression model.

Predictors^a^	β	95% CI	η^2^	*P* value
(Intercept)	463.47	104.65 to 822.29	N/A^b^	.004
MVPA^c^	.33	–0.40 to 1.06	0.18	.47
BBS^d^	.11	–1.16 to 1.37	0.01	.67
Short FES-I^e^	–15.70	–24.22 to –7.19	0.24	<.001
BMI	–1.24	–6.40 to 3.92	<0.01	.67
Age	.02	–3.61 to 3.65	<0.01	.99

^a^*R*^2^/*R*^2^ adjusted: 0.357/0.288.

^b^Not applicable.

^c^MVPA: moderate to vigorous physical activity.

^d^BBS: BTrackS Balance System.

^e^FES-I: Fall Efficacy Scale-International.

**Table 4:** Incremental Shuttle Walk Test multivariable regression model.

Predictors^a^	β	95% CI	η^2^	*P* value
(Intercept)	1106.67	554.88 to 1658.47	N/A^b^	<.001
MVPA^c^	1.36	0.24 to 2.48	0.46	.02
BBS^d^	–.66	–2.60 to 1.28	0.04	.49
Short FES-I^e^	–18.79	–31.88 to –5.70	0.21	.006
BMI	–9.30	–17.24 to –1.37	0.07	.02
Age	–4.80	–10.38 to 0.78	0.06	.09

^a^*R*^2^/*R*^2^ adjusted: 0.563/0.517.

^b^Not applicable.

^c^MVPA: moderate to vigorous physical activity.

^d^BBS: BTrackS Balance System.

^e^FES-I: Fall Efficacy Scale-International.

**Table 5:** Gait speed component of the Short Physical Performance Battery multivariable regression model.

Predictors^a^	β	95% CI	η^2^	*P* value
(Intercept)	6.15	2.71 to 9.59	N/A^b^	.001
MVPA^c^	0	–0.00 to 0.01	0.25	.17
BBS^d^	0.00	–0.01 to 0.01	<0.01	.64
Short FES-I^e^	–.14	–0.22 to –0.06	0.28	.001
BMI	0	–0.05 to 0.05	<0.01	.94
Age	–.02	–0.06 to 0.01	0.04	.19

^a^*R*^2^/*R*^2^ adjusted: 0.435/0.375.

^b^Not applicable.

^c^MVPA: moderate to vigorous physical activity.

^d^BBS: BTrackS Balance System.

^e^FES-I: Fall Efficacy Scale-International.

## Discussion

### Principal Findings

This study examined associations between several measures of physical performance and general physical health of older adults, including physical activity, static balance, FoF, and BMI. We found several significant correlations between PPTs and physical health measures and further explored these with multivariable regression models evaluating the health measures that are drivers of each PPT.

The full correlation analysis showed significant correlations (Figure 1) between most physical activity and PPTs, as well as the subjective measure of FoF (FES-I). By characterizing multiple measures of physical performance in the same sample, this study is the first to our knowledge to evaluate and compare the comprehensive value of 3 commonly used PPTs (SPPB, 6MWT, and ISWT). Among each test, the SPPB showed the greatest magnitude and consistency of significant correlations. Additionally, we note the similarity of significant correlations between objectively measured physical activity and subjectively measured FoF, emphasizing the value of subjectively collected measures in older adults.

The multivariable regression models further evaluated the comprehensive value and relevance of each PPT to the physical activity, balance, FoF, and BMI of older adults. The complete SPPB model (Table 2) was both predicted by more variables and characterized by a higher adjusted *R*^2^ value than the models predictive of gait function only (SPPB-G, ISWT, and 6MWT), indicating a greater value in holistic testing. Performance on the SPPB was significantly predicted by the FESI-I; MVPA; and in the absence of the FES-I, static balance. Designed to assess lower extremity functioning, the SPPB includes measures of standing balance, 4-meter gait speed, and the time needed to rise from a chair 5 times. This multipart test is easy to perform and evaluates several parts of daily physical functioning, indicated by the static balance test, a field walking test, and a sit-to-stand test included in the protocol. As such, the SPPB was a more comprehensive measure of older adults’ physical health, evidenced by its associations with MVPA and static balance. Separating the SPPB-G provided a foundation upon which to compare the gait-based PPTs. In Table 5, the gait speed on the 4-meter course was predicted by the FES-I. Very similarly, the 6MWT (Table 3) was only predicted by the FES-I, suggesting that performance on this test is more related to the effect of subjective FoF and its impact on willingness to participate in physical activity. Furthermore, the 6MWT is limited by several factors, including variable standardization of testing and the learning effect, where repeated administration influences performance on the test [48-50]. The 6MWT is focused on lower limb mobility as a PPT [25], and as such, may be more of an indicator of gait performance and variables related to mobility rather than overall health. This focus on mobility is observed in the relationship between 6MWT performance and its only significant predictor, the FES-I.

The ISWT was similar to the 6MWT in its aim of assessing gait; however, the ISWT is performed incrementally and externally paced. As such, it was expected to be more integrative of overall health, supported also by a higher magnitude of correlation coefficients for the ISWT than for the 6MWT. We found that the ISWT was predicted by 3 components of overall health in older adults: MVPA, BMI, and the FES-I (Table 4). While the ISWT was more parsimonious in nature than the SPPB, as shown by its multiple predictors, disadvantages include the intensity and fatiguing nature of the ISWT. The similarity of the ISWT to the 6MWT validated its link with FES-I scores, although this has not been validated in the literature for the ISWT. Additionally, the *R*^2^ values for the ISWT were more similar but not as large as the complete SPPB, and larger than the values for the SPPB-G and 6MWT, indicating its possible superiority as a comprehensive PPT. The implications of such a finding are its increased use as a standardized tool in geriatric clinician visits or preferential use of a more robust measure when evaluating gait. The broader impact of using the ISWT relates to that it is cost-effective and relatively user-friendly, not requiring significant training to administer.

### Comparison to Previous Work

Our finding of associations between physical activity and FoF is consistent with previous literature [21-23] indicating the effectiveness of PPTs in evaluating overall physical health. Among the specific PPTs, the relationship between the 6MWT and FoF is also supported by previous literature, which has found that performance on the 6MWT has previously been associated with both physiological fall risk [51] and more subjective responses to FoF, as measured by the FES-I [52]. However, this study found a lack of other significant predictors of performance on the 6MWT, which could contribute to its decreased potential for assessing the overall health of older adults in research and outpatient settings. The multiple predictors of the ISWT are more difficult to justify based on previous literature, which shows mixed results between physical activity and ISWT performance. One study among patients with heart failure has reported significant correlations between performance on the ISWT and physical activity levels [26]. However, another study in patients with chronic obstructive pulmonary disease did not find a relationship between physical activity and ISWT distance [53]. The relationship of the ISWT with BMI is notable, in that it is indicative that the more incremental approach to the walk test may be representative of overall body composition and mass. This finding has previously been reported as the result of increased exertion during the ISWT than during the 6MWT, evidenced by a higher peak heart rate [54,55]. However, the administration of the ISWT involves an externally set pace and is typically used for adults with chronic obstructive pulmonary disease; this may limit the applicability of this test for older adults with impairments, as shown by studies demonstrating decreased walking distance on the ISWT compared to the self-paced 6MWT for adults with disabilities [56].

### Strengths and Limitations

First, this study was limited by a small sample size to represent several variables of overall health. This was evident in the power analysis with a large, anticipated effect size. However, the depth of data collected on each participant is a clear strength that reflected the contribution of this work to the body of literature on PPTs. Each participant completed 3 PPTs, physical activity monitoring for several days, static balance testing, an FoF questionnaire, and BMI measurement. In doing so, this study allowed for direct comparisons of PPTs conducted in the same sample. Second, the study sample consisted of predominantly female participants (41/53, 77%). While this is representative of the population of community-dwelling older adults who attend community centers, it is an additional factor that may impact the results. To address this, separate ANOVAs and Kruskal-Wallis tests were conducted to evaluate the effect of sex and age and include them as appropriate in the analysis.

The results of this study emphasized the value of the SPPB as an indicator of overall physical health in older adults. As this population grows in size, it is critical to determine optimal standards of physical health assessment. In identifying the SPPB as an optimal indicator, the authors not only validated its use in both identifying older adults at higher risk for falls and adverse health events but also set a standard for future research in older adults. Additionally, while the 6MWT and ISWT are both field walking tests, a comparison between these 2 tests provided clarification on the response of the ISWT to measures beyond its primary variable of interest, aerobic capacity. By consolidating various measures of PPT into a single, more comprehensive test, experimental and epidemiological studies are more easily compared and reproduced.

### Future Directions

To expand this work, further studies should validate these findings in older adult samples from different communities to evaluate the external validation, as well as clinical differences over longitudinal data collection. Additionally, the mechanisms behind how each of the physical health measures affects the PPTs remains unknown. Interdisciplinary work can evaluate the multifaceted drivers of physical performance and their physiological mechanisms. Understanding these may provide further support and additional interventions for older adults.

### Conclusions

This study found that the SPPB was more comprehensive and responsive to overall physical health compared to the 6MWT and ISWT in older adults. In particular, the SPPB was predicted by both physical activity levels and FoF. It assessed balance, gait speed, and lower extremity strength through its inclusion of standing balance tests, walking, and chair stands. In contrast, field walking tests, like the 6MWT and ISWT, were more limited in their associations. These findings suggested that the SPPB should be standardized as the optimal physical performance measure in geriatric research and care, as it allows earlier identification of those at risk of functional decline. However, the ISWT may still provide value in revealing limitations at higher exertion capacities that are not apparent with the SPPB. More comparative research is warranted to develop the best assessment battery for evaluating multifaceted geriatric health.

## Appendix

Multimedia Appendix 1 Supplementary analyses investigating the significance of each linear regression model for the physical performance tests (SPPB, SPPB-G, 6MWT, and ISWT) without fear of falling as a predictor. 6MWT: 6-minute walk test; ISWT: Incremental Shuttle Walk Test; SPPB: Short Physical Performance Battery; SPPB-G: gait component of the Short Physical Performance Battery.

## Abbreviations

6MWT: 6-minute walk test
BBP: BTrackS Balance Plate
BBS: BTrackS Balance System
FES-I: Fall Efficacy Scale-International
FoF: fear of falling
ISWT: Incremental Shuttle Walk Test
LPA: light-intensity physical activity
MVPA: moderate to vigorous physical activity
PPT: physical performance test
SB: sedentary behavior
SPPB: Short Physical Performance Battery
SPPB-G: gait component of the Short Physical Performance Battery

## Data Availability

The datasets generated during or analyzed during this study are available in the Open Science Framework repository Comparison of Physical Performance Tests in Older Adults [57].
