# Supplementary material for: Common Physical Performance Tests for Evaluating Health in Older Adults: Cross-Sectional Study
Source: Interact J Med Res. 2024 Nov 29;13:e53304. doi: 10.2196/53304 (PMC11645506; doi:10.2196/53304)
Supplement: Multimedia Appendix 1 [file ijmr_v13i1e53304_app1.docx]

|  | **SPPB** | | |
| --- | --- | --- | --- |
| *Predictors* | *Estimates* | *CI* | *p* |
| (Intercept) | 13.83 | 5.54 – 22.12 | **0.002** |
| MVPA | 0.03 | 0.01 – 0.04 | **0.002** |
| BBS | -0.03 | -0.06 – -0.00 | **0.033** |
| BMI | 0.01 | -0.11 – 0.13 | 0.900 |
| Age | -0.06 | -0.14 – 0.02 | 0.119 |
| Observations | 53 | | |
| R^2^ / R^2^ adjusted | 0.395 / 0.344 | | |

|  | **SPPB-G** | | |
| --- | --- | --- | --- |
| *Predictors* | *Estimates* | *CI* | *p* |
| (Intercept) | 6.50 | 2.69 – 10.31 | **0.001** |
| MVPA | 0.01 | 0.00 – 0.02 | **0.039** |
| BBS | -0.00 | -0.02 – 0.01 | 0.748 |
| BMI | -0.00 | -0.06 – 0.05 | 0.958 |
| Age | -0.04 | -0.08 – -0.01 | **0.022** |
| Observations | 53 | | |
| R^2^ / R^2^ adjusted | 0.287 / 0.227 | | |

|  | **6-minute walk test** | | |
| --- | --- | --- | --- |
| *Predictors* | *Estimates* | *CI* | *p* |
| (Intercept) | 501.92 | 99.06 – 904.78 | **0.016** |
| MVPA | 0.67 | -0.13 – 1.46 | 0.098 |
| bbs | -0.44 | -1.82 – 0.95 | 0.529 |
| bmi | -1.60 | -7.40 – 4.20 | 0.581 |
| Age | -2.20 | -6.05 – 1.65 | 0.256 |
| Observations | 53 | | |
| R^2^ / R^2^ adjusted | 0.168 / 0.099 | | |

|  | **Incremental Shuttle Walk Test** | | |
| --- | --- | --- | --- |
| *Predictors* | *Estimates* | *CI* | *p* |
| (Intercept) | 1152.68 | 561.52 – 1743.84 | **<0.001** |
| MVPA | 1.77 | 0.60 – 2.93 | **0.004** |
| bbs | -1.31 | -3.34 – 0.72 | 0.201 |
| bmi | -9.74 | -18.25 – -1.23 | **0.026** |
| Age | -7.45 | -13.10 – -1.81 | **0.011** |
| Observations | 53 | | |
| R^2^ / R^2^ adjusted | 0.486 / 0.443 | | |
